# Concomitant Rupture of Hydatid Cyst of Liver in Hepatic Duct and Gallbladder: Case Report

**DOI:** 10.4021/gr215e

**Published:** 2010-07-20

**Authors:** Imtiaz Wani, Younis Bhat, Naveed Khan, Farooq Mir, Saima Nanda, Omar J Shah

**Affiliations:** a Department of Surgical Gastroenterology, SKIMS Srinagar, Kashmir, India; b Department of Radiodiagnosis, SKIMS Srinagar, Kashmir, India

**Keywords:** Hydatid, Rupture, Gallbladder, Hepatic duct

## Abstract

Hydatid cyst liver rupture into the biliary tree may involve the common hepatic duct, lobar biliary branches, the small intrahepatic bile ducts or rarely the gallbladder. Rupture can be occult or frank. A frank intrabiliary rupture of hepatic hydatid cyst is a rare but serious event. The authors are reporting a case of concomitant rupture of hydatid cyst of liver into right hepatic duct and the gallbladder. A 50-year-old female patient who presented with acute cholangitis was confirmed as a case of intrabilary rupture on ultrasonography, endoscopic retrograde cholangiopancreatography and magnetic resonance cholangiopancreatography. Rupture of hydatid cyst of liver in right hepatic and the gallbladder was confirmed on surgery. Suture repair of cystobiliary fistula, choledochoduodenostomy with cholecystectomy was done.

## Introduction

A myriad of complications of hydatid cyst of liver may occur. The complications may include rupture, infection, or anaphylaxis. Rupture of hydatid cyst of liver is the most common complication. Liver hydatid can rupture in any part of biliary system but the communication with the hepatic bile ducts is most common. Rupture between a hepatic hydatid cyst and the gallbladder is rare [1]. An elevated pressure inside hydatid cyst leads to rupture and most often communicating with biliary system. This can be small communication or frank rupture. However, frank rupture into the biliary tree occurs in only 5–15% of cases [2]. Biliary communication is due to incorporation of biliary radicles into the pericyst of hydatid liver. Small rupture remains occult or asymptomatic. Later in time rupture of the hydatid cyst into biliary tree produces symptoms and signs of obstructive jaundice, or sometimes an acute cholangitis occurs. Modalities of investigation for confirming diagnosis are ultrasound abdomen (USG), computed tomography scan (CT Scan) abdomen, magnetic resonance cholangiopancreatography (MRCP) and endoscopic retrograde cholangiopancreatography (ERCP). ERCP is both diagnostic as well as therapeutic and is considered the gold standard in management of intrabiliary rupture of hydatid cyst of liver. Occult fistula closes of its own while as in the treatment of large rupture, internal drainage procedure has been suggested to those who do not respond to endoscopic intervention.

## Case Report

**Figure 1:**
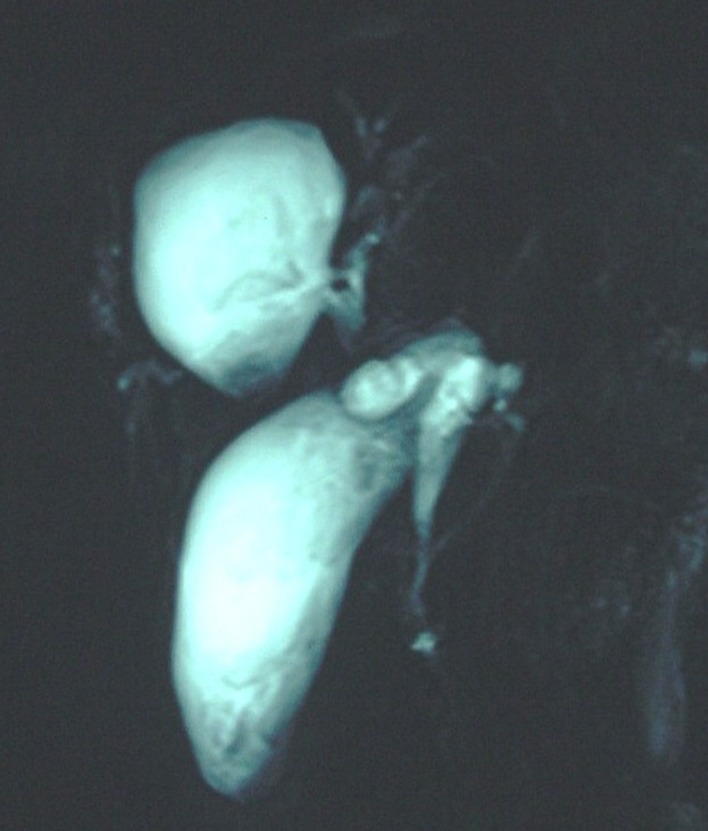
Magnetic resonance cholangiopancreatography revealed 4.5 cm liver cyst in segment IV and V with internal echoes and irregular hyperintensities inside direct communication with right hepatic duct. Common bile duct was dilated with internal debris seen inside. Gallbladder was distended showing irregular linear intensities inside.

A 65-year-old female presented with abdominal pain and jaundice of 17 days duration. After 10 days she had fever with chills and high colored urine. General physical examination revealed yellowish discoloration of sclera. Systemic examination was normal. Abdominal examination revealed tenderness in right hypochondrium and epigastrium. Liver function test parameters were bilirubin of 9.57 mg/dl, AST 52 U/L, and ALP 407 U/L. Ultrasonography abdomen revealed cystic lesion in liver with internal echogenic areas in right lobe of liver anteriorly, the gallbladder distended with thickened wall (acalcolous cholecystitis) with echogenic material (membranes) within it. Common bile duct was dilated with internal debris and linear echogenic areas (membranes) present in right hepatic duct as well as in common bile duct suggestive of intrabilary rupture of hydatid cyst of liver. Upper gastrointestinal endoscopy revealed stomach containing lot of bilious fluid with erythematous papilla with tongue like projection from papilla ruptured hydatid membrane. ERCP showed swollwen papilla, dilated common bile duct, with ruptured membranes present, the right intrahepatic duct was seen communicating with cyst cavity. Wide papillotomy was done and plastic stent 10F × 10 cm was placed across papilla. MRCP revealed 4.5 cm liver cyst in segment IV and V with internal echoes and irregular hyperintensities inside seen in direct communication with right hepatic duct. Common bile duct (CBD) was dilated with internal debris seen inside. Gallbladder was distended showing irregular linear intensities inside (Fig. 1).

The patient had repeat ERCP after four weeks revealing ruptured membranes and swollen papilla. ERCP cholangiogram revealed tube in cyst cavity with ruptured membranes present inside.

**Figure 2:**
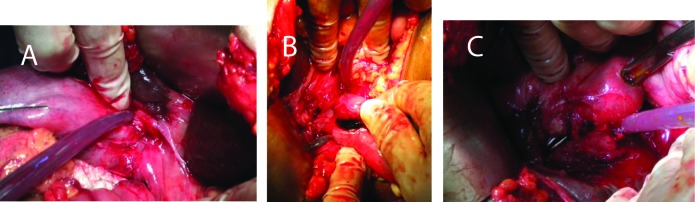
(A-C) A small fistula measuring 1 cm was seen between liver hydatid cyst and right hepatic duct and another which had fistula measuring 0.5 cm width of gallbladder.

The patient had exploratory laparotomy with peroperative findings revealing gallbladder placed vertically with short cystic duct with neck abutting hydatid cyst of liver with thickened walls. Atretic hydatid liver cyst containing infected bile with ruptured membranes. A small fistula measuring 1 cm was seen between liver hydatid cyst and right hepatic duct. Another fistula measuring 0.5 cm was found with the gallbladder (Fig. 2 A-C). Suture repair of fistula was done with evacuation of ruptured membranes from liver hydatid. Ruptured membranes with stent inside were seen after choledochotomy. Evacuation of cyst contents from liver cyst and CBD was done with choledochoduodenostomy. Cholecsytectomy was done with gallbladder containing ruptured membranes and cystic duct being patent. Histopathology of gallbladder revealed features of acalculous cholecystitis. She was discharged with Albendazole therapy. Follow-up was uneventful.

## Discussion

Spontaneous rupture into the biliary tract is the commonest complication of the liver hydatid. The causative factors for the rupture are trauma, infection or pressure from the progressively increasing size of cyst. Rupture may occur in biliary tree, thorax, peritoneum or subcutaneously. About 90% of liver hydatid cysts which rupture are communicating with biliary channels [3]. Hydatid cysts of the liver exert pressure on the surrounding parenchyma. Due to higher pressure in the cyst, the cysts eventually leak into small bile ducts or perforate into large ones. Rupture is most likely to occur in centrally located cyst with a high intracystic pressure up to 80 cm H_2_O [4]. According to Lewall and McCorkell, the cyst rupture can occur in three clinical forms: contained, communicating and direct [5]. Contained rupture occurs when the cyst contents are confined within the pericyst. Communicating rupture defines tearing of the pericyst and evacuation of cyst contents into the biliary tract or bronchioles. Direct rupture describes complete tear of the cyst wall and spillage of the cyst contents into the peritoneal or pleural cavity.

Rupture occurs in the right duct in 55-60% of cases, in the left duct in 25-30%, and rarely in the confluence or gallbladder (as in the present case) [6]. Fistula formation with the gallbladder is a very rare entity [7]. Typically, the fistulous communication is not discovered until surgery, although in some patients it is found at radiology.

Biliary obstruction is reported to occur in 5-17% of cases after rupture of hepatic hydatid [8]. It has been reported that if the cystobiliary opening is larger than 5 mm, cystic content migration into the biliary tract would occur in 65% of the cases [9]. Obstructive jaundice occurs in 57% to 100% of cases following intrabiliary rupture, especially when the rupture occurs into the large bile ducts thus emptying the contents into the biliary tract [4]. When rupture into the biliary tract occurs, the cystic fluid escapes into the biliary tract with daughter cysts; or ruptured membranes discharged into the common bile duct, causing biliary colic, obstructive jaundice, and possibly liver abscess [10, 11]. This communication between the hydatid cyst cavity and the biliary tree may produce intermittent or progressive obstructive jaundice. Other presentations are right upper abdominal pain (82%), fever (70-90%), acute cholangitis (20-37%), abdominal lump (22-39%), and rarely with acute pancreatitis, liver abscess or septicaemia; or in 5% to 6% it may be asymptomatic. Acute cholecystitis following bilary rupture occurs rarely [5, 12].

Delayed diagnosis and treatment of intrabilary rupture of liver hydatid is associated with serious morbidity (19.44-43.03%) and mortality (1.8- 4.5%) [5, 13]. Prompt diagnosis and treatment is essential in such cases. Sepsis and hepatic failure are major causes of mortality.

Ultrasound abdomen is the most commonly employed initial investigation. In frank communicating rupture, the cyst becomes smaller, and undulating membranes may be seen within it. Extrahepatic biliary dilatation is a constant feature. Echogenic or non-echogenic material without posterior acoustic shadowing is seen in the biliary tree, suggestive of sludge and daughter cysts. Direct communication was visualized in only 20% of cases. On CT abdomen visualization of detached undulating membranes and calcification of the cyst wall can be present. A dilated CBD with low attenuation intraluminal material suggests the presence of hydatid sand and cysts in the CBD. An interrupted area of the cyst wall proximal to a dilated duct may be identified as representing the site of communication. Cyst wall discontinuity, a direct sign of rupture, is seen in only 75% of cases [10]. CT can demonstrate high attenuation material passing through the defect of the cystic wall and filling up the intrahepatic biliary radicles or CBD [2]. The accuracy of CT combined with ultrasound was near 100% in cases with uncomplicated intrabiliary rupture [4]. HIDA can be helpful in doubtful cases with cystobiliary communication where ultrasound and CT are not conclusive [14]. The usual findings are a photopenic area in the liver in initial images, which gradually fills up in delayed images, indicating bile leak into the cyst, although it cannot document the exact nature of communication. The MRCP finding in ruptured hydatid cyst can be direct or indirect and is considered useful in intrabiliary rupture of liver hydatid [15]. A breach in the low intensity rim of the cyst wall with extrusion of cyst contents is a direct sign, while increased echogenicity, fluid levels, presence of air and changes in signal intensity are indirect signs on MRCP. ERCP is the gold standard in confirming biliary tract involvement. On ERCP, a swollen ampulla of Vater may be seen, with hydatid material protruding out. Dilated ducts with debris and daughter cysts may appear as radiolucent filling defects. Irregular leaf-like material that changes shape with changes in pressure differentiates this condition from other causes of obstructive jaundice [4]. A small cystobiliary communication cannot always be excluded by ERCP and needs to be actively sought during surgery [16].

Presurgical use of albendazole or mebendazole in echococcoosis infestations facilitates surgery by reducing intracystic pressure and by reducing the risk of recurrence after surgery or endoscopic evacuation of a ruptured cyst into biliary tree [8]. The localization of the cyst in the liver as well as the localization of the intrabiliary rupture is important in the strategy of the treatment. Endoscopic sphincterotomy with extraction of retained of ruptured membranes or daughter cyst in CBD is an alternative treatment for intrabiliry rupture [17]. Endoscopic treatment has a success rate of 80-90% in patients without having any surgery. Patients who had surgery for hepatic hydatid disease, endoscopic modalities offered are endoscopic sphincterotomy and nasobiliary drainage in patients with biliary fistula, balloon or bougie dilatation and stenting in patients with biliary stricture and ES with balloon extraction in patients with residual material within bile duct [18]. In such cases the overall success rate is 70-86% with a rate of fistula closure of 81% in 10-20 days [18, 19].

Liver hydatid require pericystectomy, the evacuation and drainage or resection. A waiting period of 2 months for spontaneous closure has been recommended in small fistula for most patients having small and occult fistulas, or fistulas closing within 2 weeks [20]. In large fistula openings (diameter of 5 mm or more) should definitely be sutured [21]. Suture of cysto biliary fistula using intraoperative cholangiography can be done. In large biliocystic ruptures, application of an internal drainage in addition to biliary tract suture is the most appropriate approach. Surgical procedures performed on large intrabiliary rupture include Roux-n-y cystojejunostomy, sutured fistula + tube drainage, sutured fistula + Omentoplasty, tube drainage + Omentoplasty, CBD drainage by a T-tube intubation. Drainage of the fistula in accordance with the technique of Perdrono’s internal transfistulary drainage of residual cavity method is another treatment, with relatively low complication rates [9]. Accurate pre- and intra-operative diagnosis and permanent drainage of the biliary tree by a wide choledochoduodenostomy are important to reduce morbidity and mortality [22]. In cases with Oddi stenosis a transduodenal sphincterotomy or choldedochoduodenostomy should be considered [23].

However surgery is associated with many post operative complications including persistent post operative biliary drainage, infection of residual cavity, sinus formation, recurrence or dissemination. Persistent postoperative external bile drainage is a serious complication, occurring in 3.8% to 27.5% of cases, that often necessitates reoperation [3, 24, 25].

In conclusion, concomitant intrabiliary rupture in hepatic duct and gallbladder is rare to see. ERCP is useful in diagnosis. Surgery can give final diagnosis.
